# Bioinformatics and machine learning approaches to explore the biomarkers in fatty acid degradation linked to osteoarthritis

**DOI:** 10.3389/fimmu.2026.1778565

**Published:** 2026-04-16

**Authors:** Jian Li, Jinpeng Wei, Hua Wu, Haihu Hao

**Affiliations:** 1 Department of Orthopedics, Third Hospital of Shanxi Medical University, Shanxi Bethune Hospital, Shanxi Academy of Medical Sciences, Tongji Shanxi Hospital, Taiyuan, China; 2 Department of Orthopedics, Tongji Hospital, Tongji Medical College, Huazhong University of Science and Technology, Wuhan, China

**Keywords:** bioinformatics, biomarkers, fatty acid degradation, machine learning, osteoarthritis

## Abstract

**Background:**

Fatty acid degradation (FAD) plays a crucial role in maintaining cellular energy homeostasis, with its dysfunction serves as an important pathological basis for the progression of various diseases. However, the specific regulatory mechanisms of this process in osteoarthritis (OA) remain to be further elucidated. This study aims to identify potential FAD-associated biomarkers and to investigate the role and potential mechanisms of FAD in OA.

**Methods:**

OA-related datasets and FAD-associated genes were retrieved from publicly accessible databases. Multiple bioinformatics methods were employed to reveal the potential connections among the aforementioned genes. Screening FAD-associated differentially expressed genes highly correlated with OA (hub OA-FADEGs) using machine learning methods. Single-sample gene set enrichment analysis (ssGSEA) was employed to characterize immune cell infiltration in OA and to explore their correlations with FADEGs. Additionally, scatter plots were used to evaluate the diagnostic efficacy of hub OA-FADEGs. Finally, enrichment analysis of hub OA-FADEGs and their corresponding therapeutic drugs was performed using the Drug Signatures Database (DSigDB).

**Results:**

Machine learning algorithms were applied to screen for hub OA-FADEGs, identifying APOD, COL1A1, SULF1, CHI3L1, PENK, and ADM as genes that are significantly upregulated or downregulated in OA samples. These results were subsequently verified by qRT-PCR. Furthermore, the aforementioned genes all exhibit strong diagnostic efficacy for OA. Ultimately, we identified 28 therapeutic drugs that may target hub OA-FADEGs using DSigDB.

**Conclusion:**

Based on comprehensive bioinformatics analysis, this study proposes that 6 key hub OA-FADEGs, including APOD, COL1A1, SULF1, CHI3L1, PENK, and ADM, could serve as potential diagnostic biomarkers for OA and highlights their regulatory roles in disease progression. These findings provide novel insights into the metabolic pathogenesis underlying OA.

## 1 Introduction

The chronic degenerative joint disorder, osteoarthritis (OA), is pathologically defined by the progressive loss of articular cartilage, sclerosis of subchondral bone, and osteophyte formation (1). With a global burden exceeding 654 million people, OA leads to persistent joint pain, stiffness, and functional disability, substantially impairing quality of life and contributing to significant socioeconomic costs (2). Current clinical management remains predominantly symptomatic and sequential, as no available therapy effectively reverses structural joint deterioration, highlighting the imperative for early intervention (3). While osteoarthritis has conventionally been attributed to mechanical wear-and-tear, recent research underscores its complex pathophysiology involving intertwined biomechanical, biochemical, and metabolic disturbances (4–6). Notably, evidence of tissue-specific metabolic reprogramming in articular cartilage and synovium has advanced the paradigm of OA also as a metabolic joint disease (7). In this context, therapeutic targeting of cellular energy metabolism has emerged as a promising disease-modifying strategy, particularly in the early phases of OA pathogenesis.

Fatty acids, fundamental components of cell membranes and various lipid molecules, are primarily degraded through the mitochondrial β-oxidation pathway (8). This process occurs within the mitochondrial matrix and serves as the central energy-generating metabolic route for the breakdown of long-chain fatty acids and enabling highly efficient ATP production (9). Physiologically, the process of fatty acid degradation (FAD) is a tightly regulated and highly ordered process, which is essential for maintaining energy homeostasis and cellular function. Dysregulation of this process underlies multiple metabolic disorders such as obesity, type 2 diabetes, and non-alcoholic fatty liver disease, wherein impaired β-oxidation leads to lipid accumulation, lipotoxicity, and energy imbalance, exacerbating cellular damage (10–12). In the context of OA, chondrocytes undergo metabolic reprogramming characterized by mitochondrial dysfunction and reduced fatty acid oxidation, resulting in intracellular energy deficit, lipid-driven toxicity, and accelerated matrix degradation (13–15). Concurrently, abnormal fatty acid metabolism promotes a pro-inflammatory eicosanoid profile, further intensifying joint inflammation and metabolic disturbance, thereby forming a vicious cycle that perpetuates OA progression (16). Based on the aforementioned mechanisms, targeted regulation of fatty acid metabolism pathways has emerged as a potential therapeutic strategy for intervening in the progression of OA, offering new research directions for metabolic regulation-based treatments of this disease.

In recent years, bioinformatics and machine learning have emerged as essential technologies for determining the pathophysiology and treatment targets of a variety of diseases (17). Accordingly, in this study, we retrieved OA-related datasets and FAD-related genes from the GEO database to identify key genes and their associated signaling pathways. Subsequently, weighted gene co-expression network analysis (WGCNA) was applied not only to identify differentially expressed genes (DEGs), but also to explore the correlations between gene modules and the disease (18). Importantly, machine learning-based feature selection algorithms were employed to effectively screen for FAD-related genes that regulate OA progression. This study reveals potential mechanistic links between FDA and OA, providing a basis for FDA-targeted strategies to enhance the precision diagnosis and treatment of OA.

## 2 Materials and methods

### 2.1 Data preprocessing

Three publicly available gene expression datasets (GSE51588, GSE57218, and GSE129147) related to OA were downloaded from the Gene Expression Omnibus database (GEO, https://www.ncbi.nlm.nih.gov/geo/). In addition, the two datasets (GSE169077 and GSE113825) used as validation sets were also obtained from GEO. The detailed information of the above datasets is provided in **Table 1**.

**Table 1:** Detailed information of the databases.

GEO accession	Tissue source	Sample size (OA / normal)
GSE51588	articular subchondral bone	40 OA / 10 Normal
GSE57218	Articular cartilage	66 samples from 33 patients (paired design: lesioned vs. preserved cartilage from the same joint)
GSE129147	Articular cartilage	10 injured / 9 healthy (non-weight-bearing)
GSE169077	Articular cartilage	6 OA / 5 Normal
GSE113825	Articular cartilage	19 OA / 11 Normal

In terms of data preprocessing and quality control, probe-to-gene symbol mappings were obtained for each GEO dataset based on the corresponding GPL platform information, using either Bioconductor annotation packages or the GPL files provided by GEO. Probe sets were re-annotated accordingly, and only probes mapping to official gene symbols were retained. In cases where multiple probes mapped to the same gene, the expression values were averaged to generate a single value per gene, resulting in an expression matrix with unique gene symbols. Prior to merging datasets, batch effects were assessed using principal component analysis and hierarchical clustering. The ComBat algorithm was then applied to correct for batch effects, thereby removing technical variability while preserving biological differences. The effectiveness of the batch effect correction was subsequently confirmed through visualization (19). With the integrated dataset, differential gene expression (DEGs) was performed using the “limma” package, applying thresholds of |logFC| > 1.00 and an adjusted p-value < 0.01. The expression patterns of the identified DEGs were then visualized via heatmap and volcano plot. For external validation of the key genes identified from the discovery cohort, GSE169077 and GSE113825 were used as independent validation datasets to evaluate their expression trends and diagnostic performance.

FAD-related genes were sourced from the GeneCards database (https://www.genecards.org/), with those exhibiting a relevance score greater than 2 selected for subsequent analysis.

### 2.2 Functional enrichment analysis

To further elucidate the biological functions of the identified DEGs, Gene Ontology (GO) enrichment analysis and Kyoto Encyclopedia of Genes and Genomes (KEGG) pathway analysis were performed. GO analysis identified significant associations of DEGs with molecular function (MF), biological process (BP), and cellular component (CC), while KEGG analysis mapped DEGs to known signaling pathway networks, suggesting their potential involvement in disease-related regulatory mechanisms (20). Next, to further capture the overall trends in pathway activity under different conditions, this study supplemented the analysis with Gene Set Enrichment Analysis (GSEA). Utilizing the GSEA software package (v1.66.0), 5 significantly activated signaling pathways were identified in the OA samples. By integrating GO, KEGG, and GSEA results, this study constructs a framework of potential molecular mechanisms in OA and systematically delineates the key pathway networks influencing its biological phenotypes.

### 2.3 WGCNA analysis

In this study, weighted gene co-expression network analysis (WGCNA) was employed for the systematic identification of functionally relevant gene modules in OA. Firstly, in order to enhance the robustness of the model, outlier gene filtering and data quality control were performed on the input dataset. Subsequently, a co-expression network was constructed based on the Pearson correlation matrix of gene expression and converted into a topological overlap matrix (TOM) to quantify the strength of functional associations between genes (21). Gene modules were identified using hierarchical clustering, with dynamic tree-cutting algorithms applied to refine module boundaries (preliminarily labeled with randomly assigned colors). Consequently, OA phenotype-significant modules were delineated, and their respective hub genes were isolated for subsequent analysis.

### 2.4 Identification of FADEGs

The “ggvenn” package was utilized to conduct an intersection analysis on differentially expressed genes (DEGs) from 3 independent datasets, FAD-related genes (relevance score>2), and the most significant module genes identified through WGCNA. This analysis ultimately identified a common gene set, which was designated as FAD-related differentially expressed genes (FADEGs). Next, we performed a visual analysis of FADEG’s expression patterns using R software and mapped their chromosomal locations. Furthermore, the association of these genes with established OA-associated genetic regions was evaluated to explore potential genomic overlaps.

### 2.5 Immune cell infiltration analysis

Immune cell enrichment analysis was performed on gene expression profiles from normal and osteoarthritic cartilage samples using the “GSVA” and “GSEABase” packages, and the results were visualized as box plots showing intergroup differences with the “ggpubr” package. Furthermore, the data were integrated and visualized using the “reshape2” and “tidyverse” packages to construct a heatmap depicting the correlation between FADEGs and immune cell infiltration levels (22). Spearman’s rank correlation analysis was systematically employed to evaluate both the strength and direction of these associations.

### 2.6 Machine learning

This study integrates three machine learning algorithms with complementary strengths: Least Absolute Shrinkage and Selection Operator (LASSO) regression, Support Vector Machine (SVM), and Random Forest (RF). LASSO regression for automated feature selection and biomarker identification, SVM for high-dimensional classification via optimal hyperplane construction, and while RF, as an ensemble learning algorithm, effectively enhances model stability and generalization capability by constructing multiple decision trees combined with Bootstrap sampling and random feature subsetting strategies (23, 24). Subsequently, the consensus of feature genes derived from the three algorithms defined the hub OA-FADEGs. Finally, we visualized the expression patterns of these hub genes using appropriate R packages.

### 2.7 Construction and evaluation of a nomogram

To systematically assess the utility of the developed model, a Decision curve analysis (DCA) was conducted using the “rms”package in R, based on the results of multivariable logistic regression. This analysis translates complex predictive modeling into a clinically interpretable tool for guiding therapeutic decisions by integrating multiple predictors and quantifying the standardized net benefit across a continuum of decision thresholds. Furthermore, the receiver operating characteristic (ROC) curve was generated using the “pROC” package, and the model’s discriminatory performance was evaluated by computing the area under the curve (AUC). When the AUC value exceeds 0.65, it is typically interpreted as indicative of satisfactory model (25).

### 2.8 Consensus clustering

A reliable clustering analysis technique, consensus clustering, was created to methodically assess the stability and repeatability of sample classification across various preset cluster numbers (k = 2–9 in this study). This approach successfully lowers random biases in individual clustering results by incorporating resampling approaches, improving the accuracy of identifying stable subtypes from biological data. Specifically, the “ConsensusClusterPlus” package in the R programming language was employed to implement the consensus clustering analysis in this study. Following subtype identification, pathway enrichment levels were assessed via Gene Set Variation Analysis (GSVA) implemented in R, focusing on both GO and KEGG pathways. Significant enrichment was defined as FDR<0.05.

### 2.9 Drug-gene interface

Drug-gene interaction datasets were acquired from the Drug Signatures Database (DSigDB; https://dsigdb.tanlab.org/). Enrichment analysis of hub OA-FADEGs, performed with the “enrichplot” and “tidytable” packages, revealed significantly enriched drug categories (26). The obtained association results were visualized using Cytoscape software. Finally, molecular docking was performed using the CB-DOCK2 online platform (https://cadd.labshare.cn/cb-dock2/php/index.php) to visualize the Predicted 3D binding interactions between identified drugs and their corresponding protein targets. Specifically, protein structures were prepared as follows: the crystal structures of human CHI3L1 (PDB ID: 8R42, resolution 2.32 Å) and human APOD (PDB ID: 2HZR, resolution 1.80 Å) were obtained from the RCSB Protein Data Bank (PDB). Due to the triple-helical structural characteristics of type I collagen, the AlphaFold-predicted structure of human COL1A1 (UniProt ID: P02452) was employed as the docking model. All protein structures were preprocessed using PyMOL 2.5 software to remove crystallographic water molecules and original ligands, followed by the addition of polar hydrogen atoms and assignment of charges; the final structures were saved in PDBQT format. For ligand preparation, the three-dimensional structures of chitosamine (CID: 439213) and retinol (CID: 445354) were downloaded from the PubChem database. Energy minimization was performed using Chem3D 20.0 software with the MMFF94 force field. Subsequently, AutoDockTools 1.5.7 was used to add hydrogen atoms, detect rotatable bonds, and assign charges, with the final ligands output in PDBQT format. Molecular docking was then performed using the CB-DOCK2 online platform with rigid docking (receptor kept rigid, ligand flexible). The docking box was set to dimensions of 20 Å × 20 Å × 20 Å with a grid spacing of 1.0 Å. The number of output conformations was set to 20, with an energy range of 5 kcal/mol. Each ligand was docked in triplicate to ensure consistency of results. Finally, redocking of the original co-crystallized ligands was performed as a positive control validation. The results showed RMSD values < 2.0 Å, confirming the reliability of the docking parameters.

### 2.10 Quantitative reverse transcription-PCR

Primary mouse chondrocytes were obtained as described previously (27, 28). The control group was cultured in DMEM/F12 medium (HyClone, USA) containing 10% fetal bovine serum (Gibco, USA).While the OA group was treated with medium supplemented with 10 ng/mL IL-1β (R&D Systems, USA). Total RNA was extracted after 24 hours of culture.

Total RNA was isolated with Trizol reagent (Thermo, USA) and reverse transcribed into cDNA using the PrimeScript RT Master Mix (TaKaRa, Japan). qRT-PCR was then performed on a Quantagene q225 fluorescence quantitative PCR system (Kubo, Beijing, China) with SYBR Green qPCR Master Mix (Beyotime, Shanghai, China). The 2^-ΔΔCt^ method was applied to calculate relative gene expression levels. All qRT-PCR assays were run with three replicates per sample. Primer sequences used in this study are listed in **Table 2**.

**Table 2:** The qRT-PCR primer sequence genes primer sequences.

Genes	Primer sequences (5′ - 3′)
*Actin*- Forward	CCACTGTCGAGTCGCGTCC
*Actin*- Reverse	ATTCCCACCATCACACCCTGG
*Apod* - Forward	CAGCATCCCATCTTTGTGCC
*Apod* - Reverse	GCTCACTGTCAGTTTCTCTCAG
*Col1a1*- Forward	GGAGAGAGCATGACCGATGG
*Col1a1*- Reverse	AAGTTCCGGTGTGACTCGTG
*Sulf1*- Forward	GGAGCTGTGTGGTCTTCTCC
*Sulf1*- Reverse	GCTGAGTTCTGGGAGCTTGA
*Chi3l1*- Forward	GGCAAGAAGCAAGACGTAGG
*Chi3l1*- Reverse	CTGCACGGCATAGTTCACATT
*Penk* - Forward	GTCAGAGACAGAACGGGTCC
*Penk* - Reverse	AGTGTGCACGCCAGGAAAT
*Adm* - Forward	AGAGCAACTCCAGCGTTACC
*Adm* - Reverse	GCGATGCTCTGATACCCTGA

### 2.11 Mendelian randomization analysis

In this study, a two-sample Mendelian randomization (MR) approach was employed to investigate the potential causal relationship between fatty acid levels and osteoarthritis. Summary-level data for total fatty acids (exposure) were obtained from the IEU OpenGWAS database (ID: met-d-Total_FA, n = 115,006 individuals of European descent). Genetic associations with osteoarthritis (outcome) were derived from a GWAS meta-analysis comprising 50,508 European individuals (10,083 cases and 40,425 controls) (ID: ebi-a-GCST005814). Single nucleotide polymorphisms (SNPs) significantly associated with total fatty acids at the genome-wide significance threshold (P < 5 × 10^-^^8^) were selected as instrumental variables (IVs). To ensure independence, SNPs in linkage disequilibrium (r² < 0.001 within a 10,000 kb clumping window) and weak instruments (F-statistic < 10) were excluded (29). Data harmonization was performed using the TwoSampleMR package to align effect alleles between the exposure and outcome datasets. The inverse-variance weighted (IVW) method was used as the primary analysis to estimate causal effects, supplemented by MR-Egger regression, weighted median, weighted mode, and simple mode methods. Sensitivity analyses, including Cochran’s Q test, MR-Egger intercept test, MR-PRESSO, leave-one-out analysis, and funnel plot inspection, were conducted to evaluate heterogeneity, horizontal pleiotropy, and the robustness of the results (30).

## 3 Results

### 3.1 Identification of DEGs in OA

**Figure 1:**
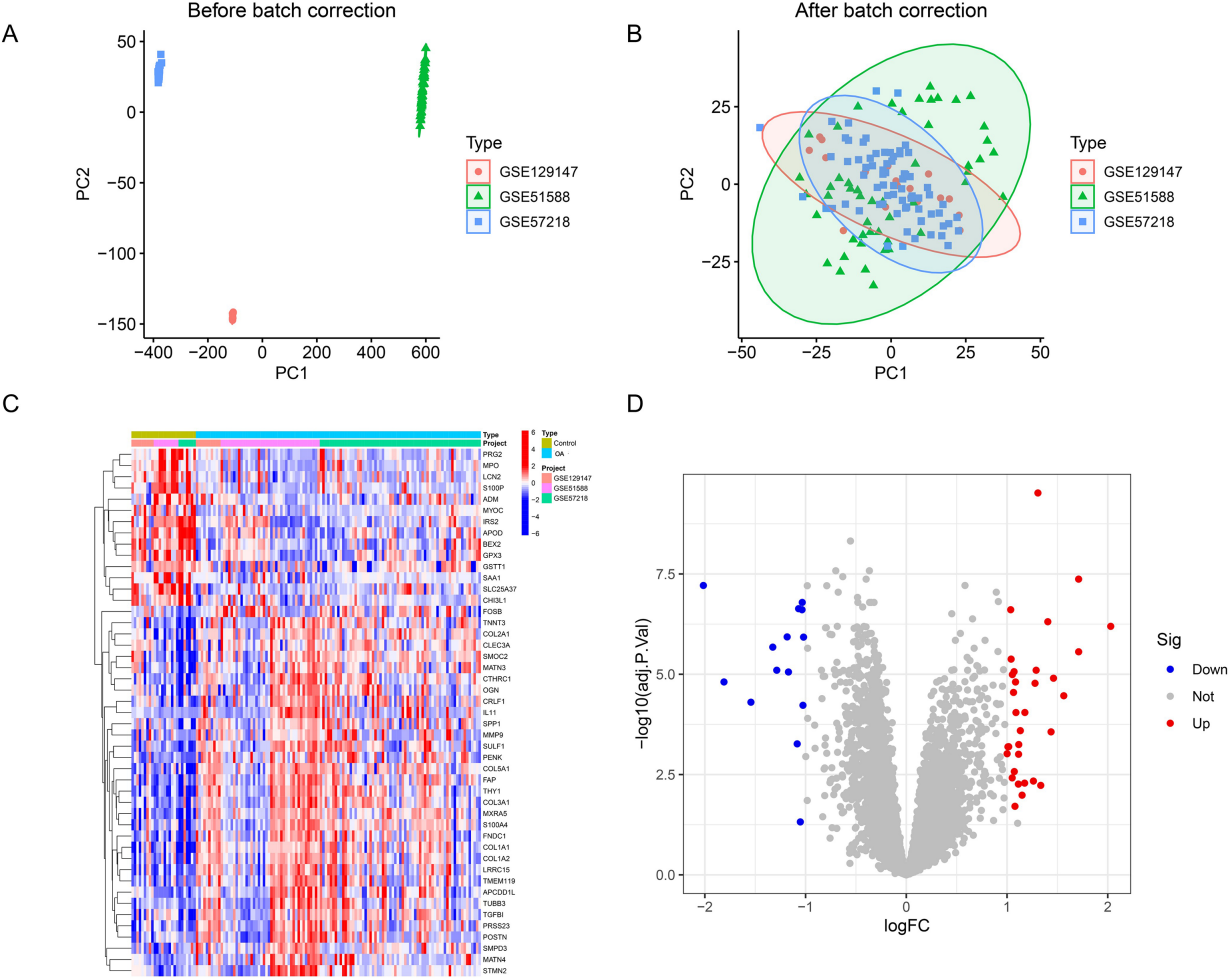
Identification of differentially expressed genes (DEGs) in OA. **(A)** Prior to batch effects removal and identification of DEGs. **(B)** Removal of batch effects and identification of DEGs. **(C)** Heatmap plot. The heatmap of DEGs displays those meeting the following significance thresholds: |log_2_FC| > 1 and FDR < 0.01. **(D)** Volcano plot. Red nodes indicate Deg upregulation, blue nodes indicate Deg downregulation, and gray nodes indicate genes with no significant differential expression.

As shown in **Figure 1A**, principal component analysis (PCA) was performed before calibration. After removal of batch effects, **Figure 1B** reveals clear clustering differences between the data, indicating that the data are suitable for further analysis. The calibrated dataset information is shown in **Supplementary Table S1**. According to the established criteria, a total of 46 DEGs were identified in the aforementioned dataset (**Figure 1C**; **Supplementary Table S2**). In addition, the volcano plot identified 14 downregulated DEGs and 32 upregulated DEGs (**Figure 1D**).

### 3.2 Functional enrichment analysis of DEGs

**Figure 2:**
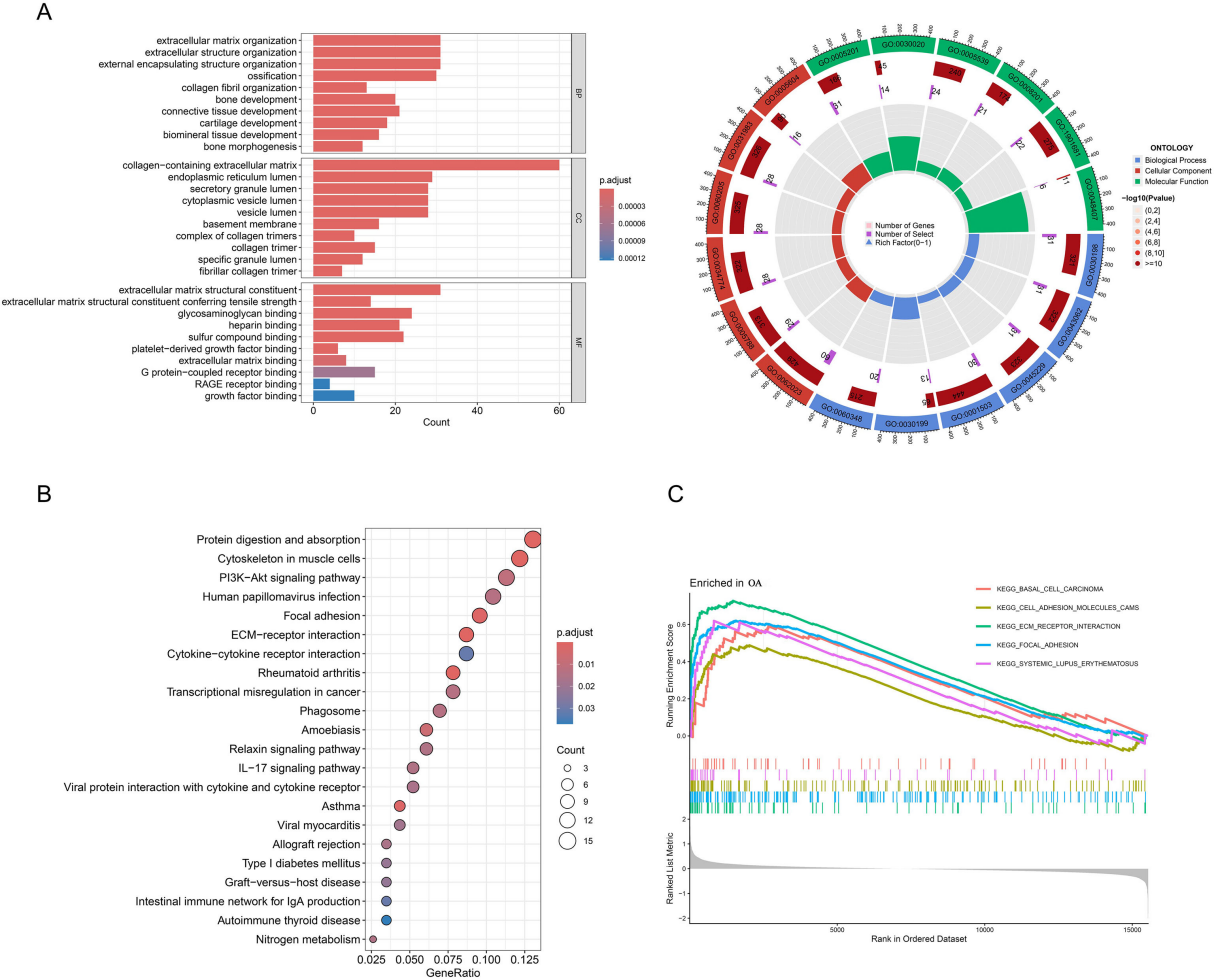
Functional enrichment analysis of DEGs. **(A)** GO enrichment analysis was performed, encompassing the three main categories: Biological Process (BP), Cellular Component (CC), and Molecular Function (MF). **(B)** Analysis of KEGG enrichment, with bubble plots displaying the most significant pathway enrichments. **(C)** Gene set enrichment analysis (GSEA), with the top 5 most significant pathways.

Subsequently, the obtained DEGs were subjected to GO and KEGG pathway enrichment analysis (**Figures 2A, B**). GO analysis revealed that these genes are significantly enriched in Extracellular matrix organization, Collagen-containing extracellular matrix, Extracellular matrix structural constituent, and etc (**Figure 2A**). KEGG analysis similarly demonstrated significant enrichment of these genes in Protein digestion and absorption, ECM-receptor interaction, and Cytokine-cytokine receptor interaction (**Figure 2B**). Moreover, GSEA pathway analysis revealed that multiple pathways, including Basal cell carcinoma, Cell adhesion molecules, ECM-receptor interaction, Focal adhesion, and Systemic lupus erythematosus, exhibited a significant upregulation (**Figure 2C**).

### 3.3 WGCNA analysis

**Figure 3:**
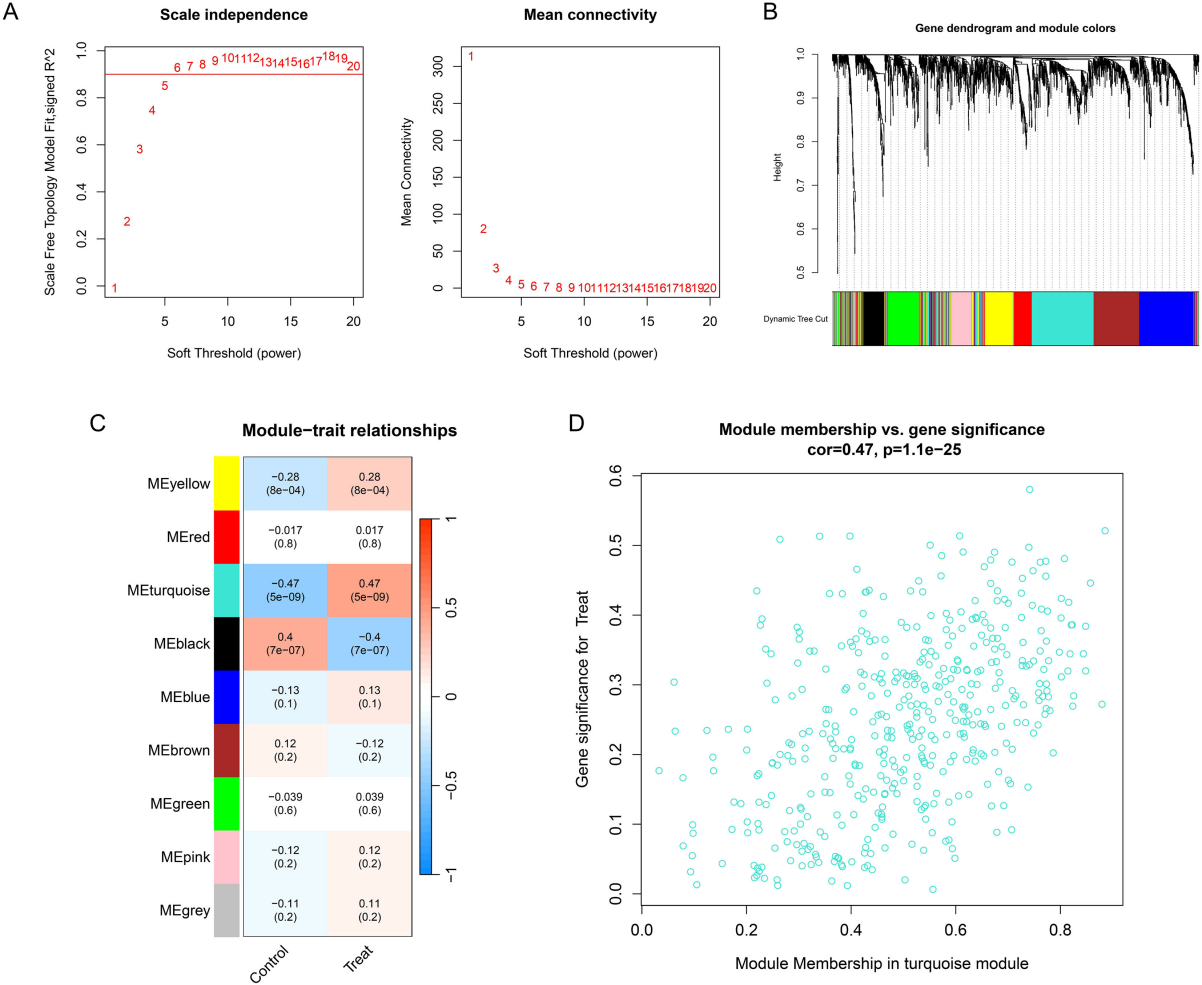
WGCNA analysis. **(A)** Network Construction with Optimal Soft Threshold (β=6, R²≥0.9). **(B)** The dendrogram displays genes clustered by variance (top 50%). Each terminal leaf represents a single gene, while the color bar below assigns them to co-expression modules. **(C)** Heatmap of module-trait relationships. Colors represent distinct co-expression modules, and the numeric values within each cell denote the correlation coefficient and p-value. **(D)** Scatter plot in turquoise module.

The top 50% of genes with the highest expression variability, comprising 2,004 genes, were selected for WGCNA analysis (**Supplementary Table S3**). An analysis network was constructed by setting the soft-thresholding power β to 6 (**Figure 3A**). A total of 9 analytical modules were identified (**Figures 3B, C**). Among them, one turquoise module exhibited the strongest correlation with osteoarthritis, containing 442 genes (correlation coefficient = −0.47, P = 5e-09) (**Figure 3C**; **Supplementary Table S3**). The correlation between gene significance (GS) and turquoise module membership (MM) for individual genes is illustrated in the scatter plot presented in **Figure 3D**.

### 3.4 Identification of FADEGs

**Figure 4:**
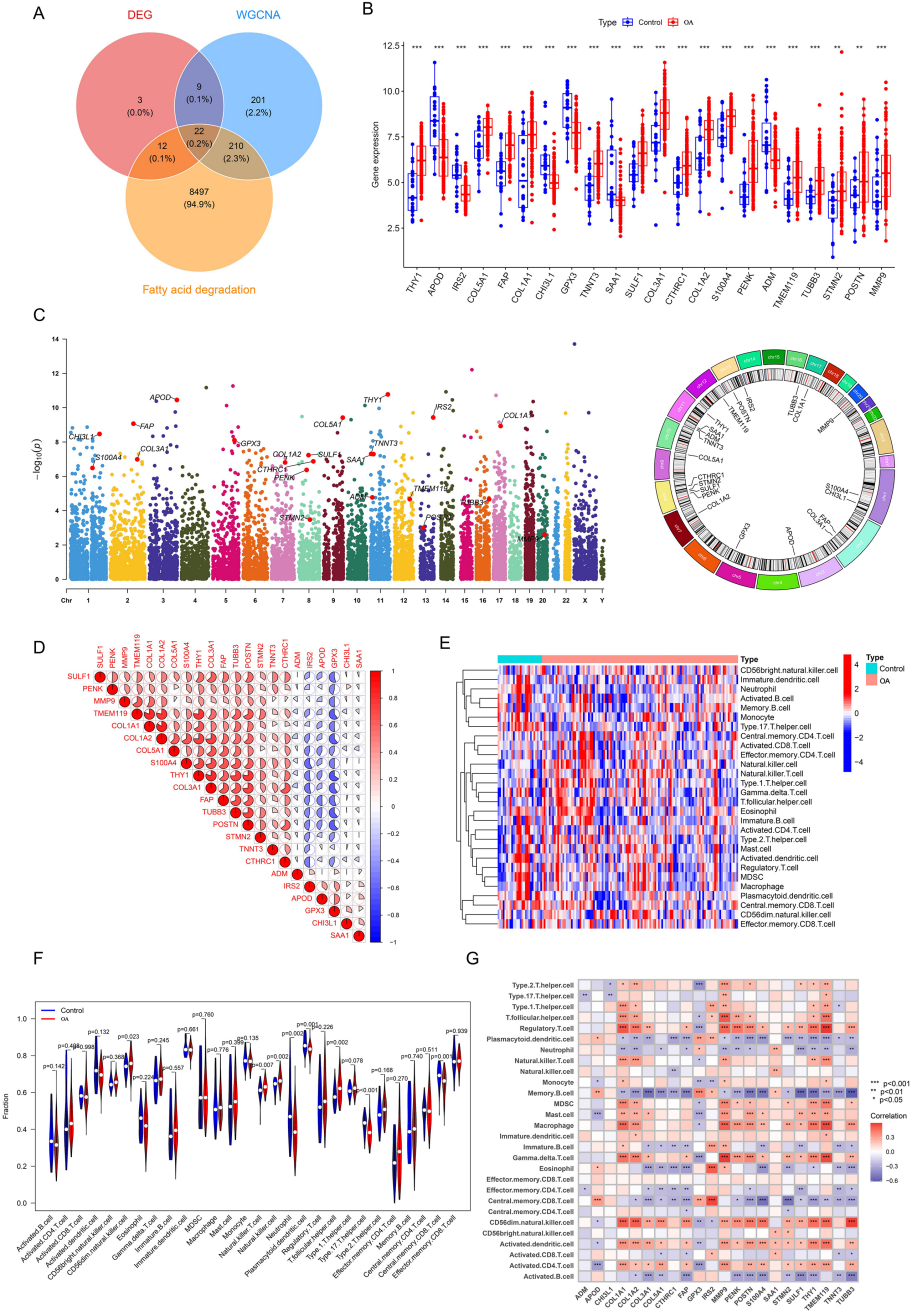
Identification of FADEGs. **(A)** DEGs, WGCNA, and Fatty acid degradation (FAD) -related genes for screening Venn diagrams of the OA- FADEGs. **(B)** Expression of 22 FADEGs in OA samples *P<0.05; **P<0.01; ***P<0.001. **(C)** Chromosome distribution of the 22 FADEGs. Statistically significant loci within chromosomal regions are highlighted. **(D)** Correlation analysis between 22 FADEGs. **(E)** Heat map of the differences in the distribution of 28 immune cells in OA. **(F)** Violin plots illustrate the differential infiltration levels of 28 immune cell types in normal versus OA cartilage samples. **(G)** Correlation analysis was performed between the 22 FADEGs and the 28 immune cell types, with statistical significance denoted as follows: *P<0.05, **P<0.01, ***P<0.001.

According to the established screening criteria, a total of 8,741 genes associated with FAD were identified (**Supplementary Table S4**). Subsequently, a cross-analysis of DEGs, WGCNA module genes (ME turquoise), and FAD-associated genes was performed using the “VennDiagram” software package. This analysis identified a total of 22 FADEGs, among which 16 genes were upregulated in OA and 6 genes were downregulated (**Figures 4A, B**). **Figure 4C** displays the chromosomal positions of each gene and the genetic clustering regions significantly associated with OA. And **Figure 4D** illustrates the potential correlations among the 22 FADEGs, which may assist researchers in identifying functionally related genes.

### 3.5 Immune cell infiltration

During the progression of OA, as illustrated in **Figures 4E, F**, there is a substantial increase in CD56dim natural killer cell, Natural killer T cell, Natural killer cell, and T follicular helper cell, accompanied by a decrease in Neutrophil, Plasmacytoid dendritic cell, Central memory CD8 T cell, and Type 17 T helper cell. Furthermore, Spearman correlation analysis was performed to explore the interactions between the 22 FADEGs and the aforementioned immune cells. The results indicate that these 22 FADEGs are associated with multiple immune cell types (**Figure 4G**). For example, COL1A1 positively correlates with CD56dim natural killer cells but negatively correlates with Neutrophil (**Figure 4G**).

### 3.6 Identification and validation of hub OA-FADEGs

**Figure 5:**
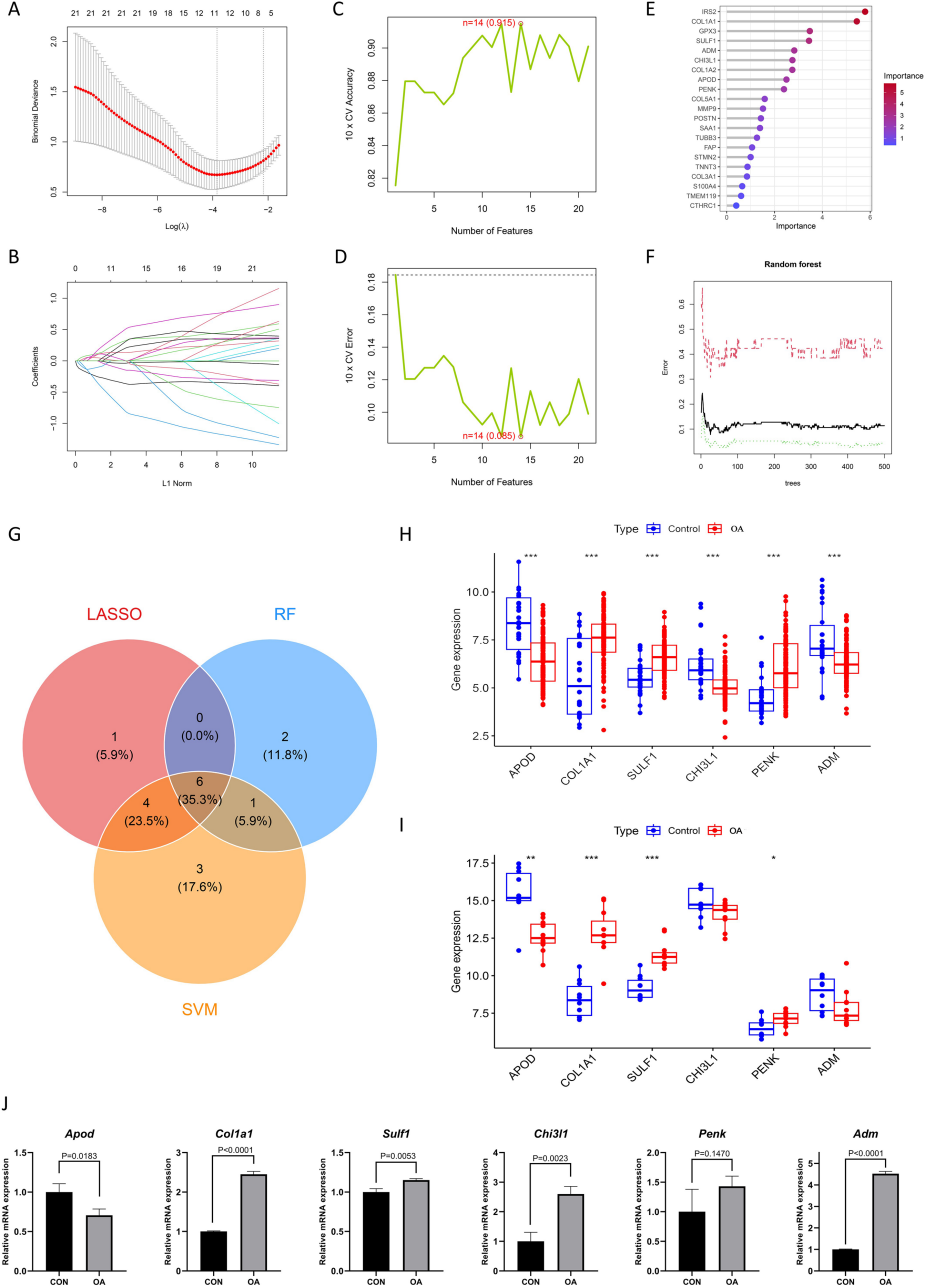
Machine learning screening hub OA- FADEGs. **(A, B)** Cross-validation for the selection of the tuning parameter in the LASSO model. **(C, D)** Maximum accuracy and minimum error plots of the SVM-RFE algorithm. **(E)** Ranking of the relative importance of hub OA- FADEGs. **(F)** Relationship between the number of random forest trees and the error rate. **(G)** LASSO, SVM-RFE and RF algorithms for screening Venn diagrams of the hub OA- FADEGs. **(H)** Expression of 6 hub OA- FADEGs in OA samples. *P<0.05; **P<0.01; ***P<0.001. **(I)** Expression of 6 hub OA- FADEGs in independent validation datasets. *P<0.05; **P<0.01; ***P<0.001. **(J)** The mRNA expression levels of 6 hub OA- FADEGs were detected by qRT-PCR.

To further validate the role of the 22 FADEGs in OA, machine learning methods were employed to screen for core genes. Three nonlinear machine learning methods were employed: LASSO (**Figures 5A, B**), SVM (**Figures 5C, D**), and RF (**Figures 5E, F**). The results of the three machine learning algorithms for recognizing hub OA-FADEGs are shown in **Supplementary Table S5**. By combining the results from the three methods above, a total of 6 hub OA-FADEGs were identified, namely APOD, COL1A1, SULF1, CHI3L1, PENK, and ADM (**Figures 5G, H**). Among these, COL1A1, SULF1, and PENK were upregulated in OA samples, while APOD, CHI3L1, and ADM were downregulated (**Figure 5H**). Additionally, the results obtained from the independent validation sets further substantiated the aforementioned conclusion (**Figure 5I**).

Then, the mRNA expression of hub OA-FADEGs was precisely quantified via qRT-PCR under different conditions (CON and OA). Specifically, the mRNA levels of *Col1a1*, *Sulf1*, *Chi3l1*, and *Adm* were significantly upregulated, while *Apod* was significantly downregulated (p < 0.05). In contrast, the expression of *Penk* showed no significant change (**Figure 5J**).

### 3.7 Hub OA- FADEGs-based risk prediction model

**Figure 6:**
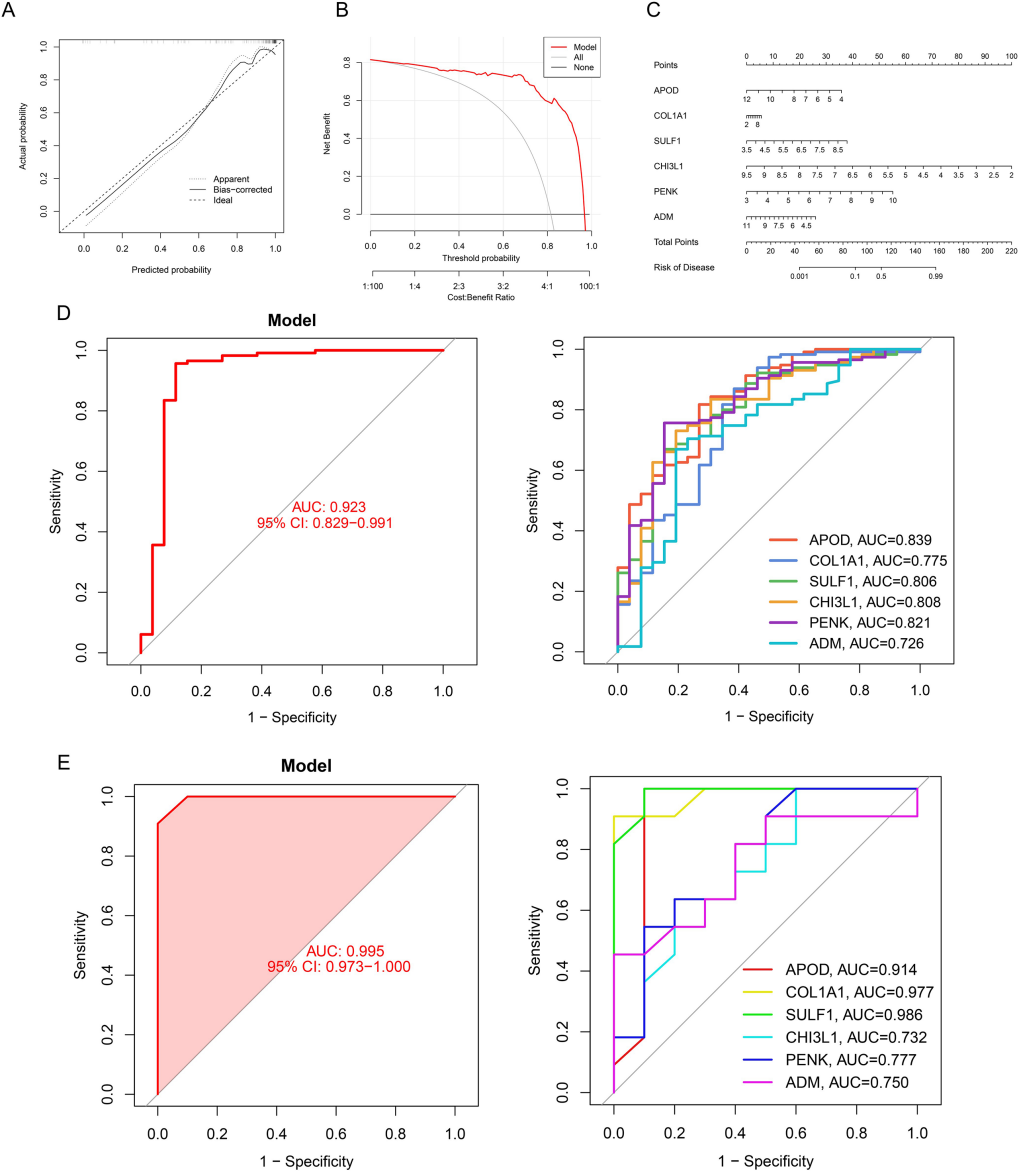
Diagnostic capability of hub OA- FADEGs. **(A)** Calibration curve for the nomogram’s predictive accuracy (closer proximity to the ideal dashed diagonal line indicates higher reliability). **(B)** Model Accuracy in Clinical Decision Curve Analysis (DCA): Distance from the Reference Line as an Indicator. **(C)** Nomogram of hub OA- FADEGs in the diagnosis of OA patients. **(D)** ROC curve analysis of hub OA- FADEGs. **(E)** ROC curve analysis of hub OA-FADEGs in independent validation datasets.

To validate the diagnostic efficacy of hub OA-FADEGs for OA, we constructed a nomogram. **Figures 6A, B** display the clinical calibration curve and clinical decision curve of the model, respectively. As shown in **Figure 6C**, the hub OA-FADEGs exhibit a variable capacity for predicting osteoarthritis risk. ROC curve analysis indicates that all 6 hub OA-FADEGs demonstrate high diagnostic value for OA (AUC > 0.65), with APOD, COL1A1 and SULF1 exhibited strong diagnostic value, as evidenced by their high AUC values (**Figure 6D**). Additionally, the results obtained from the independent validation sets further substantiated the aforementioned conclusion (**Figure 6E**).

### 3.8 Molecular subtyping of hub OA- FADEGs

**Figure 7:**
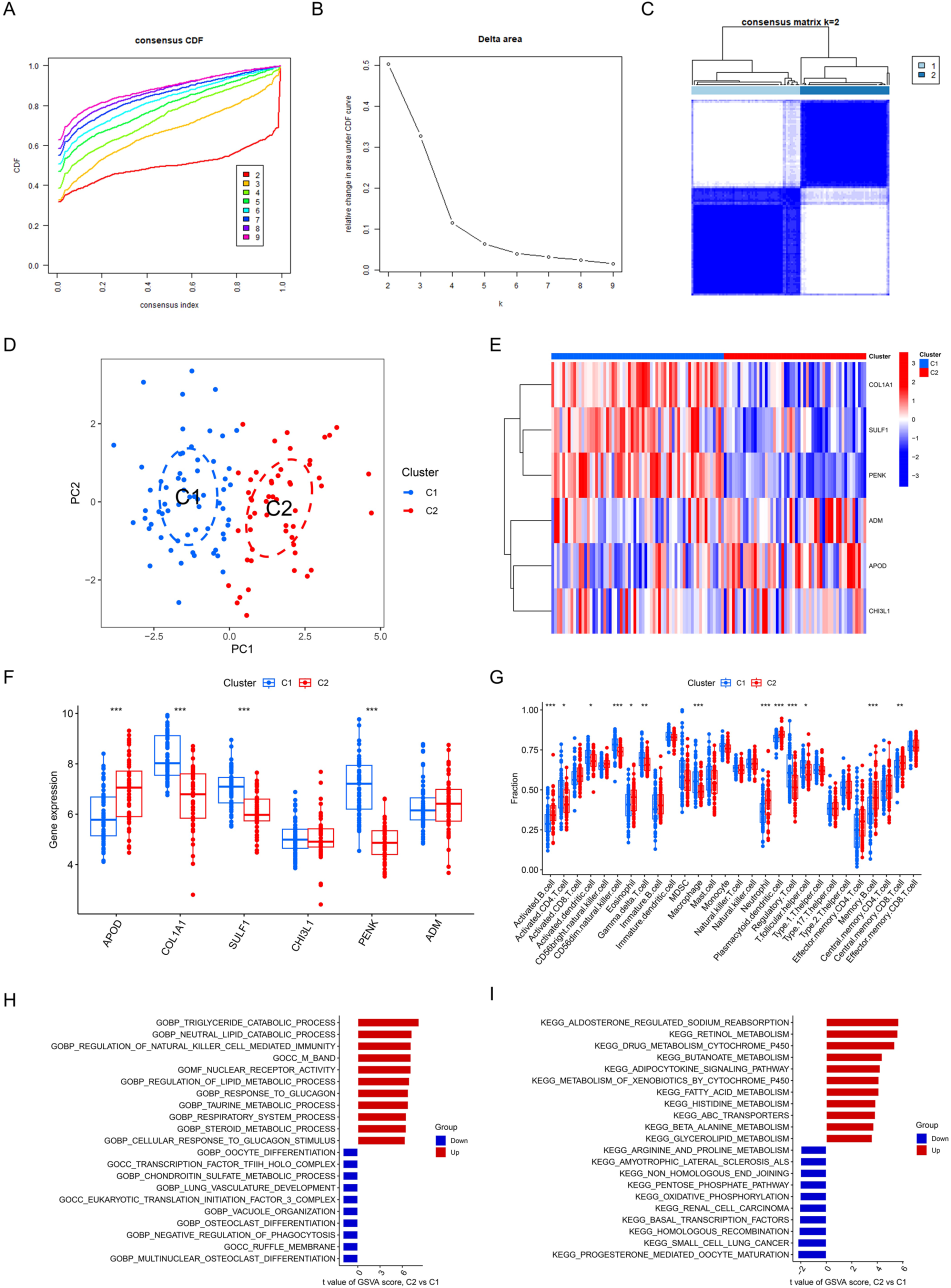
Consensus clustering of hub OA- FADEGs. **(A)** CDF curves of consensus clustering for k = 2 to 9. **(B)** Relative change in area under CDF curve. **(C)** The consensus matrix for k = 2 shows that a darker color indicates a higher consensus index. **(D)** PCA results for cluster 1 (C1) and cluster 2 (C2). **(E)** The heatmap plot of hub OA- FADEGs between C1 and C2 group. **(F)** The differential expression levels of hub OA- FADEGs between C1 and C2 group. **(G)** Violin plots showing the differences in infiltration of 28 immune cell types between C1 and C2 group. **(H)** Differences in GO enrichment between C1 and C2 group. **(I)** Differences in KEGG pathway enrichment between C1 and C2 group. *P<0.05; **P<0.01; ***P<0.001.

Next, we conducted molecular subtyping analysis. When K = 2, the cumulative distribution function (CDF) curve exhibited the flattest profile, with high clustering consistency indices within subtypes and low consistency indices between subtypes (**Figures 7A–C**). Consequently, the OA cohort was divided into two cellular subtypes, C1 and C2 (**Figure 7D**). Furthermore, DEGs analysis revealed that APOD was highly expressed in the C2 group, while COL1A1, SULF1, and PENK showed elevated expression in the C1 group. No significant differences were observed for CHI3L1 and ADM (**Figures 7E, F**). Immune infiltration analysis revealed that compared to C1, the C2 expression levels of Activated B cell, Eosinophil, Neutrophil, Plasmacytoid dendritic cell, Memory B cell, and Central memory CD8 T cell were significantly up-regulated, while that of Activated CD4 T cell, CD56dim natural killer cell, and Macrophage were down-regulated (**Figure 7G**). Furthermore, the GSVA method was employed to investigate the enrichment of GO and KEGG pathways of two subtypes. The results of GO enrichment analysis and KEGG pathway enrichment analysis are presented in **Figures 7H, I**.

### 3.9 Drug- hub OA- FADEGs interface

**Figure 8:**
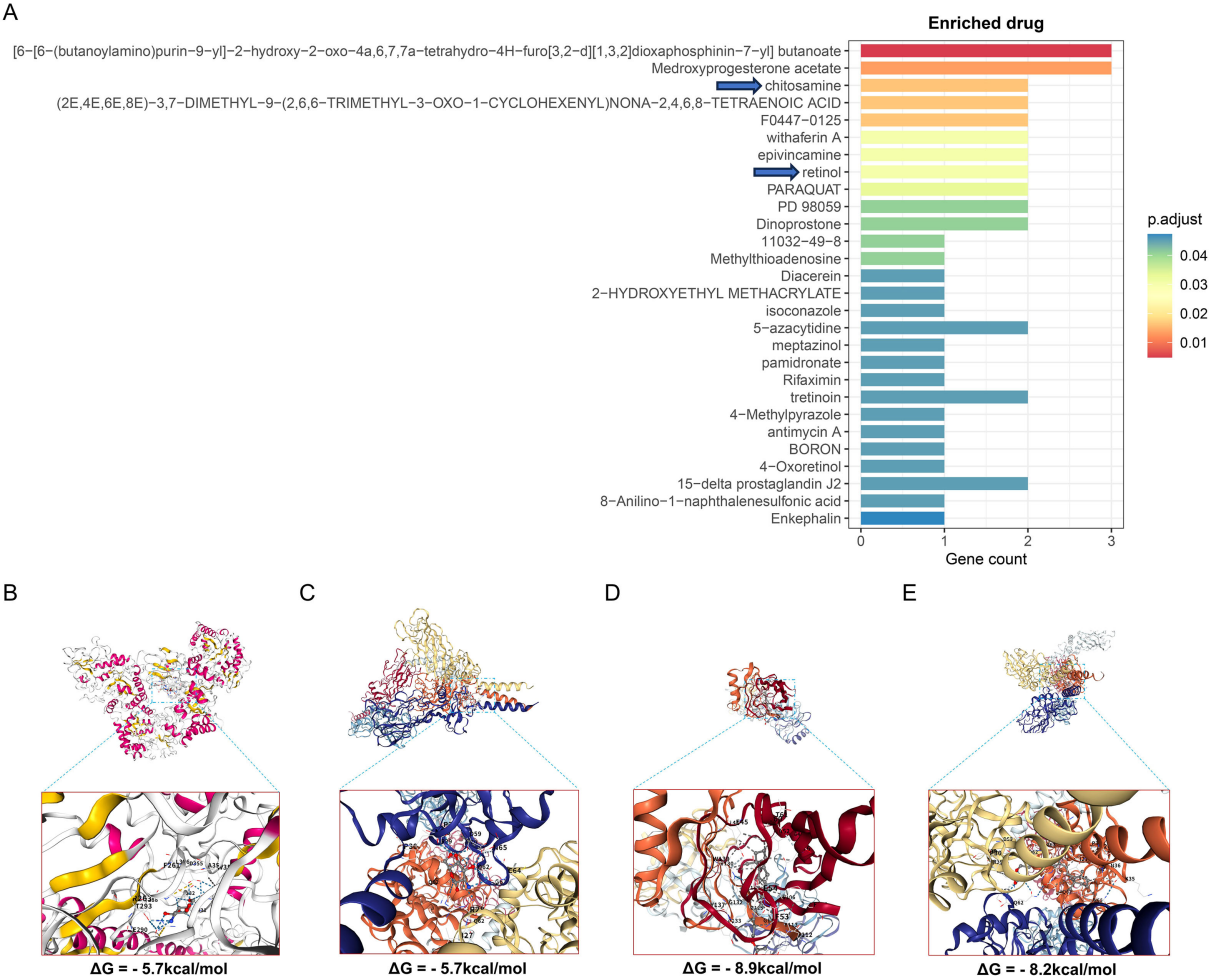
Drug enrichment and molecular docking of hub OA- FADEGs. **(A)** Enrichment relationships between hub OA- FADEGs and drugs. **(B)** The molecular docking results between chitosamine and CHI3L1. **(C)** The molecular docking results between chitosamine and COL1A1. **(D)** The molecular docking results between retinol and APOD. **(E)** The molecular docking results between retinol and COL1A1.

To identify potential therapeutic drugs targeting hub OA-FADEGs, we conducted a systematic analysis based on the DSigDB database. In this analysis, a total of 28 candidate drugs potentially targeting hub OA-FADEGs were identified. Among them, two drugs can simultaneously target three hub OA-FADEGs, twelve drugs can target two hub OA-FADEGs, and fourteen drugs each target a single hub OA-FADEGs (**Figure 8A**; **Supplementary Table S6**). Furthermore, we conducted molecular docking experiments for drugs targeting chitosamine (D-glucosamine) and retinol, respectively. The results indicate that two drugs exhibit high binding affinity for their respective targets (**Figures 8B–E**). Specifically, the docking results showed that chitosamine successfully docked into the active site pocket of CHI3L1, with a binding free energy of -5.7 kcal/mol (**Figure 8B**). Chitosamine was found to bind to the groove region of the collagen triple helix of COL1A1, with a binding free energy of -5.7 kcal/mol **Figure 8C**). Retinol was fully embedded into the hydrophobic binding pocket of APOD, exhibiting a binding free energy of -8.9 kcal/mol (**Figure 8D**). Additionally, retinol bound to the superficial groove region of the collagen triple helix of COL1A1, with a binding free energy of -8.2 kcal/mol (**Figure 8E**).

In this study, DSigDB was utilized as an initial in silico screening tool to generate research hypotheses based on drug-gene expression associations. Of note, this database primarily relies on drug-induced gene expression profiles and may not fully capture the pharmacological effects of compounds in specific tissues such as cartilage, particularly under pathological conditions involving mechanical loading or inflammation. Therefore, the screening results are insufficient for predicting *in vivo* efficacy. Given this limitation, the predicted drug effects warrant further functional validation through *in vitro* experiments using chondrocytes or ex vivo cartilage explant models.

### 3.10 Results of mendelian randomization

To investigate the potential causal relationship between fatty acid levels and OA from a genetic perspective, we performed a systematic analysis using a two-sample Mendelian randomization approach.

**Figure 9:**
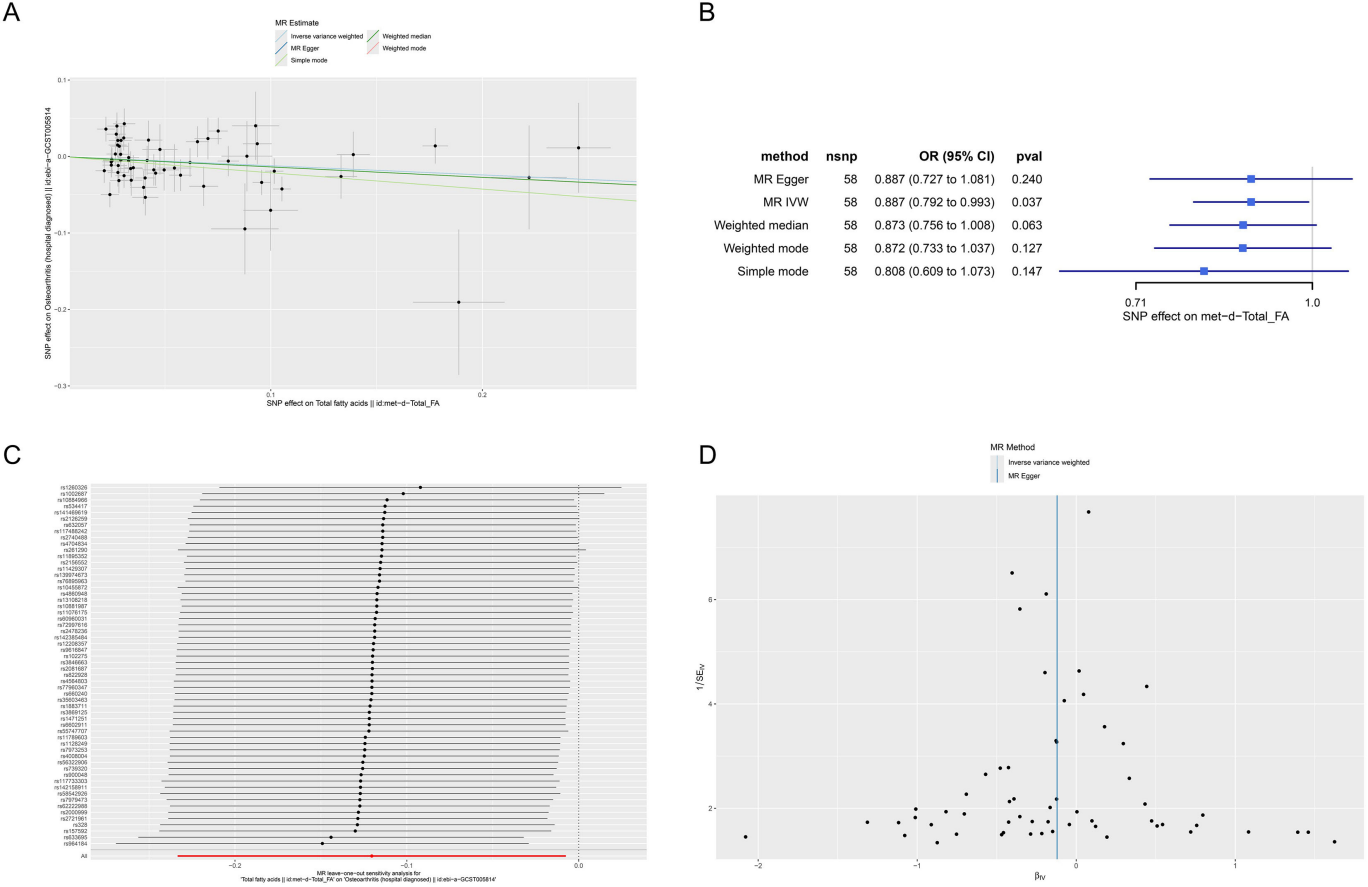
Mendelian randomization analysis of the causal relationship between fatty acid levels and OA risk. **(A)** Scatter plots showing the consistency of causal estimates across multiple MR methods. **(B)** Forest plot displaying individual SNP effects and overall MR estimates, with the IVW method indicating a significant protective effect. **(C)** Leave-one-out sensitivity analysis confirming that no single SNP drives the association. **(D)** Funnel plot demonstrating no significant directional pleiotropy.

Scatter plots were generated to display the effect estimates of each SNP as an instrumental variable on the exposure (fatty acid levels) and the outcome (OA) (**Figure 9A**). The results showed that the effect directions of various MR methods-including inverse variance weighting, MR-Egger regression, weighted median, weighted mode, and simple mode-were generally consistent, indicating good robustness of the findings. Forest plots illustrated the causal effect estimates of individual SNPs and the overall MR analysis on the outcome (**Figure 9B**). The results revealed that the overall IVW estimate indicated a significant causal relationship between genetically determined fatty acid levels and the risk of OA (P = 0.037,OR=0.887, protective factor).

Leave-one-out sensitivity analysis was conducted to assess whether any single SNP disproportionately influenced the overall causal estimate (**Figure 9C**). By systematically removing each SNP and re-performing the MR analysis, we observed that the pooled estimates from the remaining SNPs remained largely consistent with the original estimate, and all effect directions were concordant. This suggests that no single SNP drove the observed association, further supporting the robustness and reliability of the results. Funnel plots were employed to evaluate potential bias and asymmetry (**Figure 9D**). The funnel plots demonstrated a roughly symmetrical distribution, indicating no significant directional pleiotropy, thereby further validating the reliability of the findings.

In summary, the MR analysis provides genetic evidence supporting a significant causal relationship between fatty acid levels and OA risk (protective factor), reinforcing the association between FAD and OA. Sensitivity analyses confirmed the robustness of the results, and the symmetry of the funnel plots suggested the absence of substantial directional pleiotropy.

## 4 Discussion

In recent years, Emerging evidence has highlighted the potential connection between lipid metabolism and OA (31, 32). As essential precursors for endogenous lipid synthesis, fatty acids are increasingly recognized for their involvement in the pathogenesis and progression of osteoarthritis. Supporting this, prior studies analyzing serum and cartilage specimens from OA patients further demonstrated a significant association between OA progression and various lipid metabolites, including fatty acids (33, 34). For instance, Wang et al. reported that dysregulation of TGF-β/BMP signaling in OA chondrocytes induces NFIA upregulation, enhancing fatty acid synthesis and oxidation, while NFIA inhibition restored metabolic homeostasis and reduced synovitis, cartilage degeneration, and pain in obese mice, highlighting fatty acid metabolism as a potential therapeutic target for OA (32). Furthermore, recent lipidomic studies have revealed compositional alterations in human OA cartilage, including increased ceramides, decreased cholesterol esters, diglycerides, and triglycerides, as well as reduced odd-chain fatty acids (C17:0) associated with HACL1 deficiency, which collectively drive chondrocytes toward catabolic metabolism (35). These findings suggest that lipid stress and inflammatory mechanisms work together to trigger and exacerbate the development of OA (31). This study integrated bioinformatics analysis and machine learning approaches to identify 6 hub OA- FADEGs, including APOD, COL1A1, SULF1, CHI3L1, PENK, and ADM. It further systematically revealed FAD-related pathways and immune cell infiltration patterns in OA. Additionally, drug analysis targeting hub OA- FADEGs provides new insights for precision-targeted therapy in OA.

Consistent with prior reports, apolipoprotein D (APOD) expression was found to be significantly downregulated in osteoarthritis (OA) in this study (36). APOD belongs to the family of lipid transport proteins. While it does not directly catalyze FAD reactions, APOD plays a crucial role in maintaining intracellular FAD homeostasis through multiple mechanisms, including lipid transport, metabolic regulation, and modulation of oxidative stress (37). Recent years have seen growing evidence implicating APOD in the protective modulation of OA progression (38). For instance, Li et al. recently demonstrated that reduced serum APOD levels could serve as a potential diagnostic biomarker for OA (39). Functional enrichment analysis results indicate that extracellular matrix-related pathways play a significant role in the progression of OA. COL1A1 serves as the primary structural protein of the extracellular matrix. While its presence in healthy cartilage tissue is minimal, COL1A1 expression is markedly upregulated under inflammatory conditions (40). Recent studies have revealed a potential link between COL1A1 and lipid metabolism, particularly FAD (41). Research indicates that cells with high COL1A1 expression often exhibit a metabolic phenotype characterized by enhanced fatty acid β-oxidation, which may serve to meet their increased energy demands (42). Furthermore, COL1A1 has been shown to directly enhance mitochondrial fatty acid β-oxidation capacity through the activation of NF-κB and AMPK signaling pathways, thereby promoting overall FAD (42, 43). The association between SULF1 and OA was first revealed by Shuhei Otsuki et al. in 2008, who reported a significant upregulation of both SULF1 mRNA and protein expression in aged OA cartilage (44). This altered expression was suggested to contribute to abnormal chondrocyte activation through modifications in heparan sulfate proteoglycan sulfation patterns. Recent studies have further revealed that SULF1 promotes extracellular matrix deposition in the tumor microenvironment, thereby influencing tumor progression (45). Currently, the direct regulatory relationship between SULF1 and FAD remains unclarified. Based on existing evidence, we hypothesize that ECM alterations may function as an intermediary mechanism linking SULF1 expression to the modulation of FAD during OA progression.

In mouse OA chondrocytes, we observed a significant upregulation of CHI3L1 expression. However, this finding is not entirely consistent with the data trends reported in certain public databases. While historically viewed as a pro-inflammatory mediator and cytokine, CHI3L1 has more recently been implicated in fatty acid metabolism, opening a new direction of research (46, 47). Research by Di Lu et al. indicates that CHI3L1 can induce reactive oxygen species (ROS) and lipid accumulation via the TNF-α/TNF-R1 signaling pathway, leading to the accumulation of lipid peroxides (LPO) (46). In other words, CHI3L1 can directly or indirectly reprogram cellular fatty acid metabolism by regulating multiple signaling pathways, thereby influencing cellular function. Additionally, this study identified a protein potentially associated with pain perception in OA—proenkephalin precursor (PENK). A recent study employed mass spectrometry (MS) to characterize the spinal peptide profile in a rat model of osteoarthritis pain revealed significant changes in the levels of 624 peptides derived from 29 precursor hormones, including PENK (48). These findings provide new insights into elucidating the molecular mechanisms underlying OA-related pain. However, subsequent experimental validation of this study revealed no statistically significant changes in PENK expression in the OA group, which may be attributable to the fact that PENK regulation entails complex multi-systemic interactions that are not fully captured by simplified *in vitro* cellular models. Adrenomedullin (ADM), an endogenous bioactive peptide belonging to the calcitonin gene-related family and functioning as an adipokine, plays a significant role in adipocyte activity (49). Notably, a 2016 study reported significantly elevated ADM levels in knee OA patients compared to healthy individuals, with concentrations correlated with disease severity (50). Further investigations have revealed that ADM upregulates integrin activation, thus promoting cell–ECM protein adhesion (51). In accordance with the findings of the KEGG pathway analysis, DEGs linked to FAD significantly influence OA pathogenesis, largely through the modulation of extracellular matrix remodeling. Our findings thus provide new insights into the pathogenic axis connecting FAD, ECM remodeling, and OA development. However, it must be acknowledged that the inconsistency between the GEO dataset findings and the qPCR validation results for genes such as CHI3L1, ADM, and PENK. Tissue heterogeneity is a potential contributing factor to this discrepancy: the GEO data were derived from human cartilage tissue, whereas the qPCR validation was performed in mouse articular chondrocytes. Future studies employing cross-species and multi-model experimental designs are warranted to further validate the roles of these target genes in the pathogenesis of OA and to address the limitations of the current validation approach.

Accumulating evidence indicates that immune cell infiltration has emerged as a key contributor to the onset and progression of OA (52).In this study, we employed Spearman correlation analysis to reveal interactions between FADEGs and immune cells. Specifically, the genes APOD, COL1A1, SULF1, and PENK exhibited significant associations with distinct immune cells. For instance, APOD correlated with Activated CD4 T cells; COL1A1 was linked to Activated CD4 T cells, CD56dim natural killer cells, and Natural killer T cells; SULF1 showed associations with Neutrophils, Memory B cells, and Activated B cells; and PENK was related to CD56dim natural killer cells and Activated B cells. The aforementioned findings expand our understanding of the potential link between fatty acid metabolism and immune cell infiltration in OA.

This study has several limitations. First, as the data are drawn from public databases, heterogeneity in sample characteristics could introduce variability into the analytical outcomes. Second, due to limitations in the current sample size, the generalizability of certain analytical conclusions requires independent validation using larger or multicenter samples. Finally, In the initial screening, the comprehensive GeneCards database was used, incorporating over 8,000 FAD-related genes. While this approach ensured sensitivity, it also introduced a risk of false positives. Through multi-layer filtration-including differential expression analysis, WGCNA, and machine learning-6 hub OA- FADEGs were ultimately identified. Although literature review confirmed their potential association with FAD, the possibility that these genes are primarily related to OA cannot be entirely excluded. Future studies should employ more rigorous strategies, such as utilizing pathway databases (e.g., KEGG), intersecting multiple databases, and conducting colocalization analysis for further validation.

## 5 Conclusion

Overall, this study identified hub OA-FADEGs represented by APOD, COL1A1, SULF1, CHI3L1, PENK, and ADM, demonstrating their outstanding diagnostic potential for OA. Further analysis revealed intrinsic links among FAD, extracellular matrix remodeling, and immune cell infiltration, offering new insights into understanding the pathogenesis of OA. Of course, these findings require further experimental validation.

## Data Availability

The original contributions presented in the study are included in the article/**Supplementary Material**. Further inquiries can be directed to the corresponding authors.
